# Blinatumomab consolidation in children with high-risk first-relapse B-cell precursor acute lymphoblastic leukemia: final 5-year follow-up analysis of a randomized multicenter phase 3 study

**DOI:** 10.1038/s41375-025-02800-6

**Published:** 2025-11-13

**Authors:** Franco Locatelli, Carmelo Rizzari, Bernd Gruhn, Thomas Klingebiel, Rosanna Parasole, Christin Linderkamp, Christian Flotho, Arnaud Petit, Concetta Micalizzi, Cornelia Eckert, Anja Möricke, Mary Sartor, Ondrej Hrusak, Christina Peters, Vaskar Saha, Luciana Vinti, Rajendra Desai, Michael Henry, Gerhard Zugmaier, Arend von Stackelberg

**Affiliations:** 1 IRCCS Ospedale Pediatrico Bambino Gesù, Rome, Italy; 2 Catholic University of the Sacred Heart, Rome, Italy; 3 Pediatric Hematology Oncology Unit, IRCCS Foundation San Gerardo dei Tintori, Monza, Italy; 4 Department of Pediatrics, Jena University Hospital, Jena, Germany; 5 Department for Children and Adolescents, University Hospital Frankfurt, Goethe University, Frankfurt am Main, Germany; 6 Azienda Ospedaliera di Rilievo Nazionale Santobono Pausilipon, Napoli, Italy; 7 Medizinische Hochschule Hannover, Hannover, Germany; 8 Universitätsklinikum Freiburg, Freiburg, Germany; 9 Hopital Armand Trousseau, APHP.Sorbonne Université, Paris, France; 10 Istituto Pediatrico di Ricovero e Cura a Carattere Scientifico G Gaslini, Genova, Italy; 11 Charité – Universitätsmedizin Berlin, Campus Virchow-Klinikum Pädiatrie m.S. Onkologie/Hämatologie, Berlin, Germany; 12 Universitätsklinikum Schleswig-Holstein, Kiel, Germany; 13 Westmead Hospital, Sydney, NSW Australia; 14 Charles University Prague University Hospital, Prague, Czech Republic; 15 St. Anna Children’s Hospital, Children’s Cancer Research Institute, Vienna, Austria; 16 Division of Cancer Sciences, School of Medical Sciences, Faculty of Biology, Medicine and Health, University of Manchester, Manchester, UK; 17 Tata Translational Cancer Research Centre, Tata Medical Center, Kolkata, India; 18 IQVIA Inc., Thane, India; 19 Amgen Inc., Thousand Oaks, CA USA; 20 Amgen Research (Munich) GmbH, Munich, Germany; 21 Charité Universitaetsmedizin CVK Berlin, Berlin, Germany; 22 Present Address: IRCCS Istituto Giannina Gaslini, Genova, Italy

**Keywords:** Haematological cancer, Acute lymphocytic leukaemia

## To the Editor:

Allogeneic hematopoietic stem cell transplantation (alloHSCT) is the standard of care consolidation treatment in children with high-risk first-relapse B-cell precursor acute lymphoblastic leukemia (B-ALL) who achieve a second complete remission with conventional chemotherapeutic regimens [1, 2]. Blinatumomab—a CD3/CD19-directed bispecific T-cell engager (BiTE®) molecule—was shown to confer superior event-free survival (EFS) versus chemotherapy in children with high-risk first-relapse B-ALL [3]. In the primary analysis (data cutoff, July 2019) of a phase 3, open-label randomized controlled study of pediatric patients with high-risk first-relapse B-ALL who underwent re-induction and two blocks of consolidation chemotherapy, a single cycle of intravenous blinatumomab during the third consolidation improved EFS (*p* < 0.001) compared with consolidation with a third cycle of conventional chemotherapy [3]. By 2 years of follow-up (data cutoff, September 2021), blinatumomab was associated with enhanced overall survival (OS) (HR 0.34, stratified log-rank *p* = 0.002) and EFS versus conventional chemotherapy (HR 0.35, stratified log-rank *p* < 0.001) [4]. The EFS benefit was observed across patient subsets stratified by time-to-relapse, extramedullary disease, and baseline measurable residual disease (MRD) [4]. Blinatumomab achieved deep and complete remission ( < 10^−4^ leukemia cells in the bone marrow) in a higher proportion of patients versus conventional chemotherapy and led to undetectable leukemia cells in the bone marrow by polymerase chain reaction testing [4]. Here, we report long-term outcome results from the 5-year follow-up analysis (data cutoff, November 21, 2022) of this study.

In this phase 3 open-label multicenter study (NCT02393859), children (aged >28 days and <18 years) with Philadelphia chromosome–negative first-relapse high-risk B-ALL were eligible to enroll if they exhibited M1 ( < 5% morphologic leukemic blasts) or M2 ( ≥ 5% and <25% morphologic leukemic blasts) bone marrow after undergoing induction and two cycles of consolidation chemotherapy. Consolidation chemotherapy protocols have been described in detail in the earlier publication from this study group [3]. Patients were randomized (1:1) to receive either a continuous intravenous infusion of blinatumomab (15 μg/m^2^/day for 4 weeks) or a third conventional chemotherapy cycle, per the IntReALL HR 2010 protocol [5]. Children who maintained a second complete remission with M1 marrow morphology after the randomized treatment were eligible for alloHSCT. Data from all randomized patients were subject to efficacy analyses and data from all patients who had received either blinatumomab or conventional chemotherapy were included in the safety analysis. The primary efficacy endpoint was EFS, with the events of interest being any cause of mortality (including death in complete remission), second relapse, secondary malignancy, and failure to achieve/maintain complete morphologic remission at the end of the randomized treatment. Secondary endpoints included OS, cumulative incidence of relapse, MRD remission at the end of treatment ( < 10^−4^ leukemic blasts), survival rate at 100 days after alloHSCT, and incidence of adverse events. EFS rates after the third consolidation therapy were analyzed after stratification into subgroups according to age (1–9 years or other [<1 year and >9 years]), bone marrow response (M1 vs M2), MRD status (negative [<10^−3^] or positive [≥10^−3^]), sex, time from first diagnosis to relapse ( < 18 months or ≥18 months to <30 months), and extramedullary disease at relapse (present or absent). The National Cancer Institute Common Terminology Criteria for Adverse Events (CTCAE) v4.0 grading scale was used for adverse event grading.

**Fig. 1:**
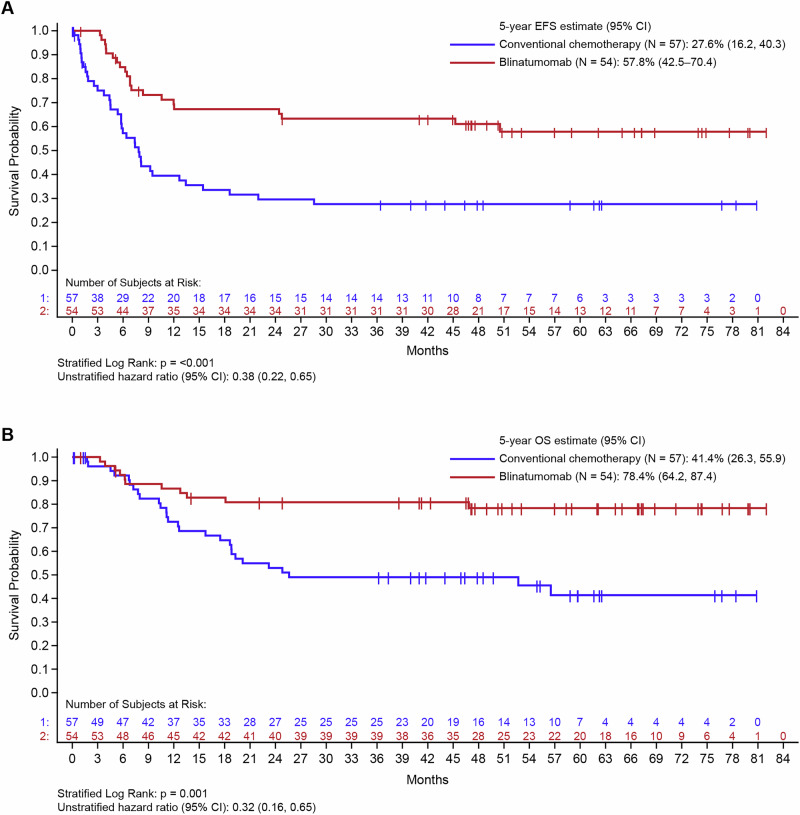
Kaplan-Meier estimates for survival outcomes in pediatric patients with high-risk first-relapse B-ALL who received blinatumomab consolidation versus conventional chemotherapy. **A** Event-free survival and **B** Overall survival. B-ALL B-cell acute lymphoblastic leukemia, CI confidence interval, EFS event-free survival, OS overall survival.

Eligible patients with B-ALL (*N* = 111), enrolled between November 2016 and August 2019, were randomized to receive either continuous intravenous infusion of blinatumomab (*n* = 54) or conventional chemotherapy (*n* = 57). Baseline characteristics, including MRD, were generally well-balanced between both treatment groups (Supplementary Table 1). Five patients randomized to the conventional chemotherapy control arm did not receive treatment (four patients’ families withdrew consent and one patient died before receiving study treatment), while all patients randomized to the blinatumomab experimental arm received treatment (Supplementary Table 1). Forty-nine (86%) patients randomized to receive conventional chemotherapy, and 52 (96%) patients randomized to receive blinatumomab completed the study regimen. After a median (range) follow-up of 51.9 (0.0–82.0) months, the EFS rate was significantly superior with blinatumomab versus conventional chemotherapy (61.1% [*n* = 33] vs 35.1% [n = 20]; hazard ratio [HR] 0.38; 95% confidence interval [CI] 0.22–0.65, *p* < 0.001). The Kaplan-Meier (KM) estimate at 60 months reflected the improvement in EFS with blinatumomab (57.8%, 95% CI 42.5–70.4) versus conventional chemotherapy (27.6%, 95% CI 16.2–40.3) (Fig. 1A). A trend towards improved EFS with blinatumomab versus conventional chemotherapy was observed across patient age stratifications of 1–9 years (HR 0.34; 95% CI 0.18–0.64, *p* < 0.001) and <1 year or >9 years (HR 0.49; 95% CI 0.18–1.33, *p* = 0.16), and EFS benefit was significant irrespective of MRD-negative (HR 0.45; 95% CI 0.23–0.88, *p* = 0.019) or MRD-positive (HR 0.28; 95% CI 0.09–0.82, *p* = 0.020) status at baseline (Supplementary Fig. 1). Blinatumomab was more effective than conventional chemotherapy in achieving higher relapse-free survival probability (63% vs 31.4%, *p* < 0.001) (Supplementary Fig. 2). Patients who received conventional chemotherapy versus blinatumomab experienced higher relapse probability irrespective of age and baseline MRD status (stratified log-rank *p* < 0.001). One patient in the blinatumomab group developed a secondary malignancy, but, as this was not a relapse, this event did not count with respect to relapse-free survival.

**Fig. 2:**
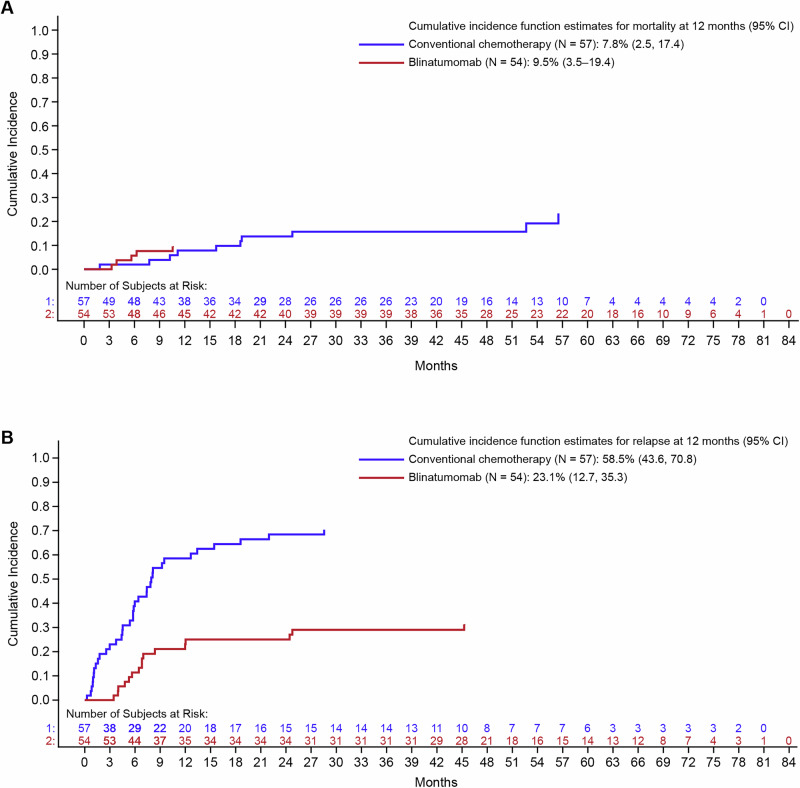
Cumulative incidence of death in pediatric patients with high-risk first-relapse B-ALL who received blinatumomab consolidation versus conventional chemotherapy. **A** Death in complete remission and **B** relapse with death due to causes other than disease (competing event) B-ALL B-cell acute lymphoblastic leukemia, CI confidence interval.

Significant improvement in OS was seen with blinatumomab versus conventional chemotherapy in the overall study population (79.6% vs 50.9%, stratified log rank *p* = 0.001). The KM estimate at 60 months, a timeframe often associated with long-term remission and potential cure, reflected the improvement in OS with blinatumomab (78.4%, 95% CI 64.2–87.4) versus conventional chemotherapy (41.4%, 95% CI 26.3–55.9) (Fig. 1B). The analyses stratified according to predefined risk groups showed improved OS for age groups 1–9 years (HR 0.29; 95% CI 0.12–0.70, *p* = 0.005) and for baseline MRD status (HR 0.37; 95% CI 0.16–0.86, *p* = 0.020 for patients who were MRD negative at baseline and HR 0.09; 95% CI 0.01–0.71, *p* = 0.022 for patients who were MRD positive at baseline). The <1 year or >9 years group showed a trend toward improved OS (HR 0.43; 95% CI 0.13–1.42, *p* = 0.16) (Supplementary Fig. 3). There was no difference between blinatumomab and conventional chemotherapy in reducing the cumulative incidence of non-relapse mortality at 12 months (9.5% vs 7.8%, Fig. 2A). Six (11.1%) patients treated with blinatumomab, and 18 (31.6%) patients treated with conventional chemotherapy died due to relapse or disease progression. Relapses with death due to causes other than disease progression were numerically lower with blinatumomab versus conventional chemotherapy (9.3% [*n* = 5] vs 17.5% [*n *= 10], difference [95% CI] −8.3 [−22.1, 5.0], *p* = 0.27; Fig. 2B and Supplementary Tables 2, 3). Three patients (3/54; 6%) in the blinatumomab group and one patient (1/57; 2%) in the conventional chemotherapy group experienced CD19-negative relapses with only one more patient in the blinatumomab group experiencing a CD19-negative relapse since the last follow-up at 44 months [4]. The proportion of patients who underwent alloHSCT was significantly higher among those who received blinatumomab versus conventional chemotherapy (94.4% [n = 51] vs 68.4% [*n* = 39], difference [95% CI] 26.0 [10.6–40.5], *p* = 0.0005). The KM estimate of median time to death after alloHSCT was 51.1 months (95% CI, 1.0–14.8) in the consolidation chemotherapy group and was not estimable in the blinatumomab group. The KM analysis of survival at 60 months after alloHSCT revealed that 79.5% (95% CI, 65.2–88.5) of patients in the blinatumomab arm were alive versus 46.6% (95% CI, 29.5% to 62.1) in the consolidation chemotherapy arm.

Treatment-emergent adverse events (TEAEs) were observed in all patients receiving blinatumomab and in 96.2% (*n* = 50) of patients receiving conventional chemotherapy. Of these, 83.3% (*n* = 45) of TEAEs in the blinatumomab arm and 78.8% (*n* = 41) of TEAEs in the conventional chemotherapy arm were related to treatment (hereafter referred to as TRAEs). No fatal TEAEs or TRAEs were observed. Compared with patients treated with conventional chemotherapy, patients treated with blinatumomab experienced a lower incidence of grade ≥3 TRAEs (16.7% vs 63.5%, *p* < 0.001). TRAEs that led to blinatumomab discontinuation occurred in 3.7% (*n* = 2) of patients. No patient receiving conventional chemotherapy discontinued treatment due to a TRAE. Given the mechanism of action of blinatumomab, cytokine release syndrome (CRS) is a known safety concern. The blinatumomab and conventional chemotherapy treatment arms had similar rates of low-grade (grade ≤2) CRS events (*n* = 2/54 [3.7%] with blinatumomab vs n = 1/52 [1.9%] with conventional chemotherapy), and all CRS events resolved with standard treatment. Furthermore, no grade ≥3 CRS was observed with either treatment arm. Neurologic adverse events were observed mainly as low-grade (grade ≤2) events (blinatumomab vs conventional chemotherapy, 57.4% vs 32.7%), with a low incidence of events of grade 3 (3.7% vs 1.9%) and grade 4 (1.9% vs 0%) severity.

Blinatumomab provided consistent EFS benefit in this phase 3 trial with respect to relapse, maintenance of complete remission, or death post-alloHSCT irrespective of baseline MRD status. The deep MRD remission previously observed with blinatumomab [6] was confirmed during this long-term follow-up analysis, with a greater proportion of blinatumomab-treated patients achieving MRD-negative status and proceeding to transplantation. In addition to CRS or the neurologic AEs previously discussed, blinatumomab demonstrated no additional safety concerns over conventional chemotherapy in terms of risk of infection, gastrointestinal disorders, or vascular disorders.

Several studies (AIEOP-BFM ALL 2017, Total XVII protocol, and JRCT2031230581) are ongoing to investigate the use of blinatumomab during consolidation in first-line therapy of pediatric B-ALL [7–9]. In particular, the AALL1731 phase 3 trial involving children with newly diagnosed standard-risk B-cell ALL, who had an average or higher risk of relapse, demonstrated that adding blinatumomab to combination chemotherapy significantly improved disease-free survival [10]. The results from the AALL1731 trial have helped establish the standard of care for consolidation therapy in first-line treatment of childhood ALL in North America. Blinatumomab treatment does not exclude the possibility of using CD19-directed chimeric antigen receptor T-cell products in case of subsequent relapse with persistence of CD19 positivity, and additional studies are needed to determine the optimal way to maximize the benefits of these therapies. As demonstrated by the updated OS data, the outcomes have significantly improved since the prior interim analysis. Notably, the plateau effect is now more pronounced, indicating a sustained long-term benefit with blinatumomab consolidation.

## Supplementary information

Supplemental Material

## Data Availability

Qualified researchers may request data from Amgen clinical studies. Complete details are available at: http://www.amgen.com/datasharing.
