# Supplementary material for: Blinatumomab consolidation in children with high-risk first-relapse B-cell precursor acute lymphoblastic leukemia: final 5-year follow-up analysis of a randomized multicenter phase 3 study
Source: Leukemia. 2025 Nov 13;40(1):230–4. doi: 10.1038/s41375-025-02800-6 (PMC12789001; doi:10.1038/s41375-025-02800-6)
Supplement: Supplementary file 1 — Supplemental Material [file 41375_2025_2800_MOESM1_ESM.docx]

**SUPPLEMENTARY MATERIAL**

**Supplementary Table 1. Baseline demographic and clinical characteristics**

| **Baseline characteristic** | **Blinatumomab**  **(N=54)** | **Conventional chemotherapy**^a^  **(N=57)** |  |
| --- | --- | --- | --- |
| **Age, years** |  |  |  |
| Median (range) | 6.0 (1-17) | 5.0 (1-17) |  |
| **Age group - n (%)** |  |  |  |
| <1 year | 0 (0.0) | 0 (0.0) |  |
| 1 to 9 years | 39 (72.2) | 41 (71.9) |  |
| ≥10 to 18 years | 15 (27.8) | 16 (28.1) |  |
| **Sex - n (%)** |  |  |  |
| Male | 30 (55.6) | 23 (40.4) |  |
| Female | 24 (44.4) | 34 (59.6) |  |
| **Race**^b^ **- n (%)** |  |  |  |
| White | 50 (92.6) | 46 (80.7) |  |
| Other | 3 (5.6) | 5 (8.8) |  |
| Asian | 1 (1.9) | 3 (5.3) |  |
| Black or African American | 0 (0.0) | 3 (5.3) |  |
| **Ethnicity - n (%)** |  |  |  |
| Hispanic/Latino | 1 (1.9) | 3 (5.3) |  |
| Not Hispanic/Latino | 53 (98.1) | 54 (94.7) |  |
| **B-cell precursor subtype - n (%)** |  |  |  |
| Pro–B-ALL | 3 (5.6) | 6 (10.5) |  |
| Pre-B-ALL | 20 (37.0) | 20 (35.1) |  |
| Common ALL | 31 (57.4) | 31 (54.4) |  |
| **Occurrence and type of any genetic abnormality - n (%)** | 20 (37.0) | 26 (45.6) |  |
| Hyperdiploidy | 6 (11.1) | 7 (12.3) |  |
| Hypodiploidy | 1 (1.9) | 0 (0.0) |  |
| t(v;11q23)/*KMT2A* rearranged | 0 (0.0) | 4 (7.0) |  |
| t(12;21)(p13;q22)/*ETV6::RUNX1* | 2 (3.7) | 3 (5.3) |  |
| t(1;19)(q23;p13.3)/*TCF3::PBX1* | 2 (3.7) | 2 (3.5) |  |
| t(5;14)(q31;32)/*IGH::IL3* | 0 (0.0) | 0 (0.0) |  |
| Other | 9 (16.7) | 10 (17.5) |  |
| **History of extramedullary disease at the time of the first relapse - n (%)** | 10 (18.5) | 15 (26.3) |  |
| Central nervous system | 11 (20.4) | 12 (21.1) |  |
| Testis | 1 (1.9) | 1 (1.8) |  |
| Other | 1 (1.9) | 3 (5.3) |  |
| **Central bone marrow assessment**^c^ |  |  |  |
| **Cytomorphology - n (%)**^d^ |  |  |  |
| M0 | 0 (0.0) | 0 (0.0) |  |
| M1 | 54 (100.0) | 54 (94.7) |  |
| M2 | 0 (0.0) | 2 (3.5) |  |
| M3 | 0 (0.0) | 0 (0.0) |  |
| Not evaluable | 0 (0.0) | 1 (1.8) |  |
| **MRD assessed by PCR value**^c^**- n (%)** |  |  |  |
| ≥10^−4^ | 10 (18.5) | 15 (26.3) |  |
| <10^−4^ | 20 (37.0) | 22 (38.6) |  |
| **MRD assessed by flow cytometry value**^c^**- n (%)** |  |  |  |
| ≥10^−4^ | 9 (16.7) | 13 (22.8) |  |
| <10^−4^ | 27 (50.0) | 24 (42.1) |  |
| **Time from the first diagnosis to relapse, mean (SD), months** | 21.88 (8.04) | 22.79 (11.92) |  |
| **Time from the first diagnosis to relapse, n (%)** |  |  |  |
| <18 months | 19 (35.2) | 22 (38.6) |  |
| ≥18 months and ≤30 months | 32 (59.3) | 31 (54.4) |  |
| >30 months | 3 (5.6) | 4 (7.0) |  |
| ALL, acute lymphoblastic leukemia; B-ALL, B-cell acute lymphoblastic leukemia; MRD, measurable residual disease; PCR, polymerase chain reaction; SD, standard deviation.  ^a^Five patients in the conventional chemotherapy control arm did not receive treatment: four patient families withdrew consent, and one patient died before receiving study treatment.  ^b^Race was reported on the electronic case report form in accordance with the guardian report. Options included American Indian or Alaska Native, Asian, Black or African American, Native Hawaiian or other Pacific Islander, White, or Other.  ^c^If a patient had MRD evaluated by both PCR and flow cytometry, the value obtained by PCR was included in the analysis. MRD was defined by the presence of at least 0.01% (ie, ≥10^−4^) ALL cells in a bone marrow specimen and predicts the likelihood of relapse.  ^d^M0: Representative bone marrow aspirate or biopsy with blasts <5%, with very low cellularity and with no regenerating hematopoiesis, M1: Representative bone marrow aspirate or biopsy with blasts <5%, with satisfactory cellularity and with regenerating hematopoiesis, M2: Representative bone marrow aspirate or biopsy with at least 5% and < 25% blasts, M3: Representative bone marrow aspirate or biopsy with at least 25% blasts. | | | |

**Supplementary Table 2. Primary and secondary endpoint outcomes**

|  | **Blinatumomab**  **(N=54)** | **Conventional chemotherapy**  **(N=57)** |
| --- | --- | --- |
| **Primary efficacy endpoint** |  |  |
| ***Event-free survival – n (%)*** |  |  |
| Events | 21 (38.9) | 37 (64.9) |
| Relapses |  |  |
| Isolated bone marrow relapse | 8 (14.8) | 14 (24.6) |
| M2 marrow after having achieved a complete remission | 3 (5.6) | 15 (26.3) |
| Combined bone marrow relapse | 3 (5.6) | 1 (1.8) |
| CNS extramedullary relapse | 2 (3.7) | 2 (3.5) |
| Extramedullary relapse at other sites^a^ | 0 (0.0) | 3 (5.3) |
| Death from any cause other than relapse | 4 (7.4)^b^ | 2 (3.5)^c^ |
| Second malignancy | 1 (1.9) | 0 (0.0) |
| **Secondary endpoints** |  |  |
| ***Overall survival – n (%)*** |  |  |
| Death from any cause | 11 (20.4) | 28 (49.1) |
| ***Subset of patients who underwent allogeneic hematopoietic stem cell transplant in second complete remission* – n (%)** | 51 (94.4) | 47 (82.5) |
| ***MRD remission by MRD status at baseline (MRD evaluable set),*  No./total evaluable (%)^d,e,f^** |  |  |
| MRD <10^−3^ at baseline |  |  |
| MRD response | 39/43 (90.7) | 26/37 (70.3) |
| MRD ≥10^−3^ at baseline |  |  |
| MRD response | 10/11 (90.9) | 1/19 (5.3) |
| ***KM estimate of mortality at 100 days after alloHSCT -* % (95% CI)** | n = 51 | n = 39 |
|  | 3.9  (1.0-14.8) | 5.1  (1.3-19.0) |
| ***Adverse events*, n (%)** | n = 54 | n = 52 |
| Incidence of TEAE (all grade) | 54 (100.0) | 50 (96.2) |
| Incidence of TEAE (≥grade 3) | 33 (61.1) | 43 (82.7) |
| Incidence of TRAE (all grade) | 45 (83.3) | 41 (78.8) |
| Incidence of TRAE (≥grade 3) | 9 (16.7) | 33 (63.5) |
| alloHSCT, allogeneic hematopoietic stem cell transplant; CI, confidence interval; CNS, central nervous system; KM, Kaplan-Meier; MRD, measurable residual disease; PCR, polymerase chain reaction; TEAE, treatment-emergent adverse event; TRAE, treatment-related adverse event.  ^a^Testicular extramedullary relapse was not observed in either group.  ^b^All events occurred after alloHSCT: hemophagocytic lymphohistiocytosis (in the context of acute graft rejection), respiratory failure due to pneumonia, hepatic failure (developing after graft-vs-host disease), and infection (in the context of graft-vs-host disease).  ^c^Causes of death included acute respiratory failure (occurring after alloHSCT) and fungal sinusitis.  ^d^MRD remission is defined as <10^−4^ blast cells. MRD remission was analyzed at the end of treatment (cycle 1, day 29) with the investigational product. Patients who were part of the MRD evaluable set and were missing postbaseline disease assessments were considered not to have achieved a response. Patients assessed included those in the MRD evaluable set who had MRD status at baseline as defined earlier and MRD response at the end of treatment (cycle 1, day 29) with the investigational product for the respective assessment methods.  ^e^The MRD evaluable set included patients for whom evaluable baseline MRD marker could be found with either PCR or flow cytometry.  ^f^For MRD status at baseline, if both a PCR and flow cytometry value was available, then the MRD PCR value was taken because PCR is more sensitive. | | |

**Supplementary Table 3. Causes of death unrelated to relapse or disease progression**

| **Cause of death, n (%)** | **Blinatumomab**  **(N=54)** | **Conventional chemotherapy**  **(N=57)** |
| --- | --- | --- |
| All | 5 (9.3) | 10 (17.5) |
| Infections | 1 (1.9) | 2 (3.5) |
| Fungal sinusitis | 0 | 1 (1.75) |
| Pneumonia | 1 (1.9) | 1 (1.75) |
| Immune system disorders | 1 (1.9) | 1 (1.75) |
| Cytokine release syndrome | 0 | 1 (1.75)^a^ |
| Hemophagocytic lymphohistiocytosis | 1 (1.9)^b^ | 0 |
| Myocardial infarction | 0 | 1 (1.75) |
| Cardiorespiratory arrest | 0 | 2 (3.5) |
| Acute respiratory failure | 0 | 1 (1.75) |
| Hepatic failure | 1 (1.9) | 0 |
| Multiorgan failure | 0 | 2 (3.5) |
| Unknown cause | 1 (1.9) | 1 (1.75) |
| B-ALL, B-cell acute lymphoblastic leukemia.  ^a^ The death due to cytokine release syndrome occurred after CAR-T infusion and was unrelated to study interventions.  ^b^The death due to hemophagocytic lymphohistiocytosis occurred after allogenic hematopoietic stem cell transplantation, in the context of acute graft rejection, and was unrelated to study interventions. | | |

**Supplementary Table 4: Institutional Review Boards(IRB)/Institutional Ethics Committees (IEC)**

| **Site**  **Number** | **Site Name** | **IRB/IEC Name** | **IRB/IEC Address** | **Regional IRB/IEC Name** | **Regional IRB/IEC Address** |
| --- | --- | --- | --- | --- | --- |
| 11001 | The Royal Childrens Hospital | The Royal Childrens Hospital Human Research Ethics Committee, | 50 Flemington Road,  Parkville, VIC, 3052, Australia |  |  |
| 11001 | The Royal Childrens Hospital | Hunter New England Human Research Ethics Committee | Lookout Road, Hunter New England Area Health Service Locked Bag 1, New Lambton, NSW, 2305, Australia |  |  |
| 11003 | Queensland Childrens Hospital | Childrens Health Services Human Research Ethics Committee | Herston Road, Level 3, Foundation Building, Herston, QLD, 4029, Australia |  |  |
| 11003 | Queensland Childrens Hospital | Hunter New England Human Research Ethics Committee | Lookout Road, Hunter New England Area Health Service Locked Bag 1, New Lambton, NSW, 2305, Australia |  |  |
| 11004 | Sydney Childrens Hospital | Hunter New England Human Research Ethics Committee | Lookout Road, Hunter New England Area Health Service Locked Bag 1, New Lambton, NSW, 2305, Australia |  |  |
| 11004 | Sydney Childrens Hospital | Sydney Childrens Hospital Network Human Research Ethics Committee | Corner Hawkesbury Road and Hainsworth Street, Westmead, NSW, 2145, Australia |  |  |
| 13001 | Universitair Ziekenhuis Leuven - Campus Gasthuisberg | Ethische Commissie Onderzoek UZ/KU Leuven | Herestraat 49, Campus Gasthuisberg, Leuven, 3000, Belgium | Ethische Commissie Onderzoek UZ/KU Leuven | Herestraat 49, Campus  Gasthuisberg, Leuven, 3000, Belgium |
| 13003 | Universite Catholique de Louvain Cliniques Universitaires Saint Luc | Ethische Commissie Onderzoek UZ/KU Leuven | Herestraat 49, Campus Gasthuisberg, Leuven, 3000, Belgium | Ethische Commissie Onderzoek UZ/KU Leuven | Herestraat 49, Campus  Gasthuisberg, Leuven, 3000, Belgium |
| 13003 | Universite Catholique de Louvain Cliniques Universitaires Saint Luc | Commission d Ethique Biomedicale Hospitalo- Facultaire de l Universite catholique de Louvain | Promenade de l'Alma 51, Boite B1.43.03,  Brussels, 1200, Belgium | Ethische Commissie Onderzoek UZ/KU Leuven | Herestraat 49, Campus  Gasthuisberg, Leuven, 3000, Belgium |
| 13004 | Universitair Ziekenhuis Gent | Universitair Ziekenhuis Gent - Ethisch Comite | Corneel Heymanslaan 10, Ingang 75, tweede  verdieping, Gent, 9000, Belgium | Ethische Commissie Onderzoek UZ/KU Leuven | Herestraat 49, Campus  Gasthuisberg, Leuven, 3000, Belgium |
| 13004 | Universitair Ziekenhuis Gent | Ethische Commissie Onderzoek UZ/KU Leuven | Herestraat 49, Campus Gasthuisberg, Leuven, 3000, Belgium | Ethische Commissie Onderzoek UZ/KU Leuven | Herestraat 49, Campus  Gasthuisberg, Leuven, 3000, Belgium |
| 13005 | Centre Hospitalier Regional de la Citadelle | Ethische Commissie Onderzoek UZ/KU Leuven | Herestraat 49, Campus Gasthuisberg, Leuven, 3000, Belgium | Ethische Commissie Onderzoek UZ/KU Leuven | Herestraat 49, Campus  Gasthuisberg, Leuven, 3000, Belgium |
| 13005 | Centre Hospitalier Regional de la Citadelle | Comite d'Ethique Centre Hospitalier Regional de la Citadelle | Boulevard du 12eme de Ligne 1, Liege, 4000, Belgium | Ethische Commissie Onderzoek UZ/KU Leuven | Herestraat 49, Campus  Gasthuisberg, Leuven, 3000, Belgium |
| 21001 | Fakultni nemocnice v Motole | Eticka komise pro multicentricke klinicke hodnoceni, Fakultni nemocnice v Motole | V Uvalu 84, Praha 5,  150 06, Czech Republic | Eticka komise pro multicentricke klinicke hodnoceni, Fakultni nemocnice v Motole | V Uvalu 84, Praha 5, 150 06, Czech Republic |
| 22001 | Rigshospitalet | Den Videnskabsetiske Komité for Region Hovedstaden | Regionsgården Kongens Vænge 2,  Hillerød, 3400, Denmark |  |  |
| 25001 | Centre Hospitalier Universitaire de Bordeaux - Hopital Pellegrin |  |  | Comite de Protection des Personnes Sud-Ouest et Outre Mer III | Place Amelie Raba Leon, Bordeaux Cedex, 33076, France |
| 25002 | Hopital Armand Trousseau |  |  | Comite de Protection des Personnes Sud-Ouest et Outre Mer III | Place Amelie Raba Leon, Bordeaux Cedex, 33076, France |
| 25004 | Centre Hospitalier Universitaire de Marseille  - Hopital de la Timone |  |  | Comite de Protection des Personnes Sud-Ouest et Outre Mer III | Place Amelie Raba Leon, Bordeaux Cedex, 33076, France |
| 25005 | Centre Hospitalier Universitaire de Nantes, Hopital Hotel Dieu |  |  | Comite de Protection des Personnes Sud-Ouest et Outre Mer III | Place Amelie Raba Leon, Bordeaux Cedex, 33076, France |
| 25006 | Institut Hematologie et Oncologie Pediatrique |  |  | Comite de Protection des Personnes Sud-Ouest et Outre Mer III | Place Amelie Raba Leon, Bordeaux Cedex, 33076, France |
| 25009 | Centre Hospitalier Regional Universitaire de Montpellier - Hopital Arnaud de Villeneuve |  |  | Comite de Protection des Personnes Sud-Ouest et Outre Mer III | Place Amelie Raba Leon, Bordeaux Cedex, 33076, France |
| 26002 | Universitaetsklinikum Wuerzburg | Ethik-Kommission der Medizinischen Fakultaet der Universität Wuerzburg | Versbacher Strasse 9,  Wuerzburg, 97078, Germany |  |  |
| 26003 | Universitaetsklinik Duesseldorf |  |  | Ethik-Kommission an der Med.Fakultät der Heinrich- Heine-Universität Düsseldorf | Heinrich-Heine-Universität Düsseldorf, 40204 Düsseldorf |
| 26005 | Medizinische Hochschule Hannover | Ethikkommission der Medizinischen Hochschule Hannover | Carl-Neuberg-Strasse 1, Hannover, 30625, Germany | Ethikkommission der Medizinischen Hochschule Hannover | Carl-Neuberg-Str. 1, 30625 Hannover |
| 26006 | Universitaetsklinikum Hamburg | Ethik-Kommission der Medizinischen Fakultaet der Universität Wuerzburg | Versbacher Strasse 9,  Wuerzburg, 97078, Germany | Ethik-Kommission der Ärztekammer Hamburg | Weidestr. 122 b, 22083 Hamburg |
| 26008 | Klinikum der Ludwig- Maximilians-Universitaet Muenchen | Ethik-Kommission der Medizinischen Fakultaet der Universität Wuerzburg | Versbacher Strasse 9,  Wuerzburg, 97078, Germany | Ethikkommission der Med. Fakultät der LMU München (196-15 mb) | Promotionsbüro, Dekanat der Medizinischen Fakultät der LMU, Bavariaring 19, 80336 München |
| 26009 | Universitaetsklinikum Jena | Ethik-Kommission der Medizinischen Fakultaet der Universität Wuerzburg | Versbacher Strasse 9,  Wuerzburg, 97078, Germany | Ethikkommission der Friedrich- Schiller-Universität Jena an der Medizinischen Fakultät (4381-03/15) | Bachstraße 18/Gebäude 1,  07740 Jena |
| 26010 | Charite Universitaetsmedizin Campus Virchow- Klinikum Berlin | Ethik-Kommission der Medizinischen Fakultaet der Universität Wuerzburg | Versbacher Strasse 9,  Wuerzburg, 97078, Germany | Ethik-Kommission Landesamt für Gesundheit und Soziales (LAGeSo) Berlin | Turmstr.21, 10559 Berlin |
| 26010 | Charite Universitaetsmedizin Campus Virchow- Klinikum Berlin | Ethik-Kommission beim Landesamt für Gesundheit und Soziales | Sachsische Strasse 28,  Berlin, 10707, Germany | Ethik-Kommission Landesamt für Gesundheit und Soziales (LAGeSo) Berlin | Turmstr.21, 10559 Berlin |
| 26012 | Universitätsklinikum Frankfurt am Main | Ethik-Kommission der Medizinischen Fakultaet der Universität Wuerzburg | Versbacher Strasse 9,  Wuerzburg, 97078, Germany | Ethik-Kommission des Fachbereichs Medizin der Goethe-Universität Frankfurt am Main | Theodor-Stern-Kai 7, Haus 1, 2.  OG, Zimmer 207-211, 60590  Frankfurt am Main |
| 26013 | Klinik fuer Kinderheilkunde und Jugendmedizin | Ethik-Kommission der Medizinischen Fakultaet der Universität Wuerzburg | Versbacher Strasse 9,  Wuerzburg, 97078, Germany | Ethik-Kommission des Fachbereichs Medizin der Goethe-Universität Frankfurt am Main | Universitätsklinikum Frankfurt, Haus 32, Theodor-Stern-Kai 7, 60590 Frankfurt am Main |
| 26014 | Universitätsklinikum Freiburg | Ethik-Kommission der Medizinischen Fakultaet der Universität Wuerzburg | Versbacher Strasse 9,  Wuerzburg, 97078, Germany | Ethik-Kommission der Albert- Ludwigs-Universität Freiburg | Ethik-Kommission der Albert- Ludwigs-Universität Freiburg |
| 32001 | Tel-Aviv Sourasky Medical Center | Institutional Helsinki Committee, Tel-Aviv Sourasky Medical Center | 6 Weizmann Street, Tel Aviv, 6423906, Israel |  |  |
| 32002 | Sheba Medical Center | Institutional Helsinki Committee, The Chaim Sheba Medical Center | 52621 Oncology Institute, Ramat-Gan, 5262000, Israel |  |  |
| 32003 | Rambam Medical Center | Institutional Helsinki Committee, Rambam Medical Center | 8 HaAliya HaShniya Street, Oncology Department, Rambam Medical Center, Haifa, 3109601, Israel |  |  |
| 33001 | IRCCS Ospedale Pediatrico Bambino Gesu | Comitato Etico dell IRCCS Ospedale Pediatrico Bambino Gesu | Viale Ferdinando Baldelli 41, Roma,  00146, Italy |  |  |
| 33002 | Azienda Ospedaliero Universitaria di Bologna Policlinico S Orsola Malpighi | Comitato Etico Indipendente di Area Vasta Emilia Centro | Via Albertoni 15,  Bologna, 40138, Italy |  |  |
| 33003 | IRCCS Istituto Giannina Gaslini | Comitato Etico Regione della Liguria | Largo Rosanna Benzi 10, Genova, 16132, Italy |  |  |
| 33004 | Fondazione IRCCS San Gerardo dei Tintori | Comitato Etico Brianza | Via Pergolesi 33,  Monza (MB), 20900,  Italy |  |  |
| 33005 | Azienda Ospedaliera di Rilievo Nazionale Santobono Pausilipon | Comitato Etico dell Azienda Ospedaliera Ospedale Cardarelli- Santobono | Via Antonio Cardarelli, 9, Napoli, 80131, Italy |  |  |
| 33006 | Azienda Ospedaliera di Padova | Comitato Etico per la Sperimentazione Dell Azienda Ospedaliera di Padova | Via Giustiniani, 1,  Padova, 35128, Italy |  |  |
| 33007 | Fondazione IRCCS Policlinico San Matteo | Comitato Etico Area Pavia Fondazione – IRCCS Policlinico San Matteo | Viale Camillo Golgi 19, Pavia, 27100, Italy |  |  |
| 33010 | Azienda Ospedaliera Citta della Salute e della Scienza Torino Ospedale Infantile Regina Margherita | Comitato Etico Interaziendale Azienda Ospedaliero Universitaria Citta della Salute e della Scienza | Corso Bramante 88, Azienda Ospedaliera Città della Salute e della Scienza di Torino Ospedale Molinette, Torino, 10126, Italy |  |  |
| 42002 | Prinses Maxima Centrum voor Kinderoncologie | Erasmus Medisch Centrum | Dr. Molewaterplein 40,  Rotterdam, 3015 GD, Netherlands |  |  |
| 42002 | Prinses Maxima Centrum voor Kinderoncologie | Centrale Commissie Mensgebonden Onderzoek (CCMO) | Parnassusplein 5, Den  Haag, 2511 VX,  Netherlands |  |  |
| 48001 | Uniwersytecki Szpital Kliniczny im Jana Mikulicza-Radeckiego we Wroclawiu | Komisja Bioetyczna przy Uniwersytecie Medycznym we Wroclawiu | ulica Pasteura 1,  Wroclaw, 50-367, Poland | Komisja Bioetyczna przy Uniwersytecie Medycznym we Wroclawiu | ulica Pasteura 1, Wroclaw, 50-  367, Poland |
| 48008 | Uniwersytecki Szpital Dzieciecy w Krakowie | Komisja Bioetyczna przy Uniwersytecie Medycznym we Wroclawiu | ulica Pasteura 1,  Wroclaw, 50-367, Poland | Komisja Bioetyczna Uniwersytetu Jagiellonskiego | Ulica Skawinska 8, 31-066 Krakow, Poland |
| 49001 | Instituto Portugues de Oncologia de Lisboa Francisco Gentil, EPE | Comissao de Etica para a Investigacao Clinica | Avenida do Brasil, 53 - Pavilhao, 17-A, Parque de Saude de Lisboa, Lisboa, 1749-004, Portugal | Comissao de Etica para a Investigacao Clinica | Avenida do Brasil, 53 - Pavilhao, 17-A, Parque de Saude de Lisboa, Lisboa, 1749-004, Portugal |
| 49002 | Instituto Portugues de Oncologia do Porto Francisco Gentil, EPE |  |  | Comissao de Etica para a Investigacao Clinica | Avenida do Brasil, 53 - Pavilhao, 17-A, Parque de Saude de Lisboa, Lisboa, 1749-004, Portugal |
| 58001 | Hospital Universitari Vall d Hebron | CEIC Hospital Universitari Vall d Hebron | Passeig de la Vall d Hebron 119-129, Institut de Recerca Ed Maternoinfantil Pl 13, Barcelona, Cataluña, 08035, Spain | CEIC Hospital Universitari Vall d Hebron | Passeig de la Vall d Hebron 119- 129, Institut de Recerca Ed Maternoinfantil Pl 13, Barcelona, Cataluña, 08035, Spain |
| 58003 | Hospital Universitario Infantil Niño Jesus | CEIC Hospital Universitari Vall d Hebron | Passeig de la Vall d Hebron 119-129, Institut de Recerca Ed Maternoinfantil Pl 13, Barcelona, Cataluña, 08035, Spain | CEIC Hospital Universitari Vall d Hebron | Passeig de la Vall d Hebron 119- 129, Institut de Recerca Ed Maternoinfantil Pl 13, Barcelona, Cataluña, 08035, Spain |
| 58004 | Hospital Universitario La Paz | CEIC Hospital Universitario La Paz | Paseo de la Castellana 261, Planta 8, Madrid,  Madrid, 28046, Spain | CEIC Hospital Universitari Vall d Hebron | Passeig de la Vall d Hebron 119- 129, Institut de Recerca Ed Maternoinfantil Pl 13, Barcelona, Cataluña, 08035, Spain |
| 58005 | Hospital Universitari i Politecnic La Fe | CEIC Hospital Universitari Vall d Hebron | Passeig de la Vall d Hebron 119-129, Institut de Recerca Ed Maternoinfantil Pl 13, Barcelona, Cataluña, 08035, Spain | CEIC Hospital Universitari Vall d Hebron | Passeig de la Vall d Hebron 119- 129, Institut de Recerca Ed Maternoinfantil Pl 13, Barcelona, Cataluña, 08035, Spain |
| 58006 | Hospital Regional Universitario de Malaga | Comite Coordinador de Etica de la Investigacion Biomedica de Andalucia CCEIBA | Avenida de la Innovacion s/n, Edificio Arena I, Sevilla, Andalucía, 41020, Spain | CEIC Hospital Universitari Vall d Hebron | Passeig de la Vall d Hebron 119- 129, Institut de Recerca Ed Maternoinfantil Pl 13, Barcelona, Cataluña, 08035, Spain |
| 58007 | Hospital Universitario Virgen del Rocio | Comite Coordinador de Etica de la Investigacion Biomedica de Andalucia CCEIBA | Avenida de la Innovacion s/n, Edificio Arena I, Sevilla, Andalucía, 41020, Spain | CEIC Hospital Universitari Vall d Hebron | Passeig de la Vall d Hebron 119- 129, Institut de Recerca Ed Maternoinfantil Pl 13, Barcelona, Cataluña, 08035, Spain |
| 63008 | Acibadem Adana Hastanesi | Ege University Medical Faculty Clinical Trials Ethics Committee | Ege Üniversitesi, Tıp Fakültesi Dekanlık Binası 2. Kat, Erzene Mah. Ankara Cad.  35100 Bornova İZMİR- TÜRKİYE |  |  |
| 65001 | Great North Childrens Hospital |  |  | East Midlands - Derby Research Ethics Committee | The Old Chapel, Royal Standard Place, Nottingham, NG1 6FS, United Kingdom |
| 65002 | Birmingham Childrens Hospital |  |  | East Midlands - Derby Research Ethics Committee | The Old Chapel, Royal Standard Place, Nottingham, NG1 6FS, United Kingdom |
| 65003 | Royal Manchester Childrens Hospital |  |  | East Midlands - Derby Research Ethics Committee | The Old Chapel, Royal Standard Place, Nottingham, NG1 6FS, United Kingdom |
| 65004 | Royal Marsden Hospital |  |  | East Midlands - Derby Research Ethics Committee | The Old Chapel, Royal Standard Place, Nottingham, NG1 6FS, United Kingdom |

**Supplementary Figure 1. Subgroup analysis for event-free survival outcomes**


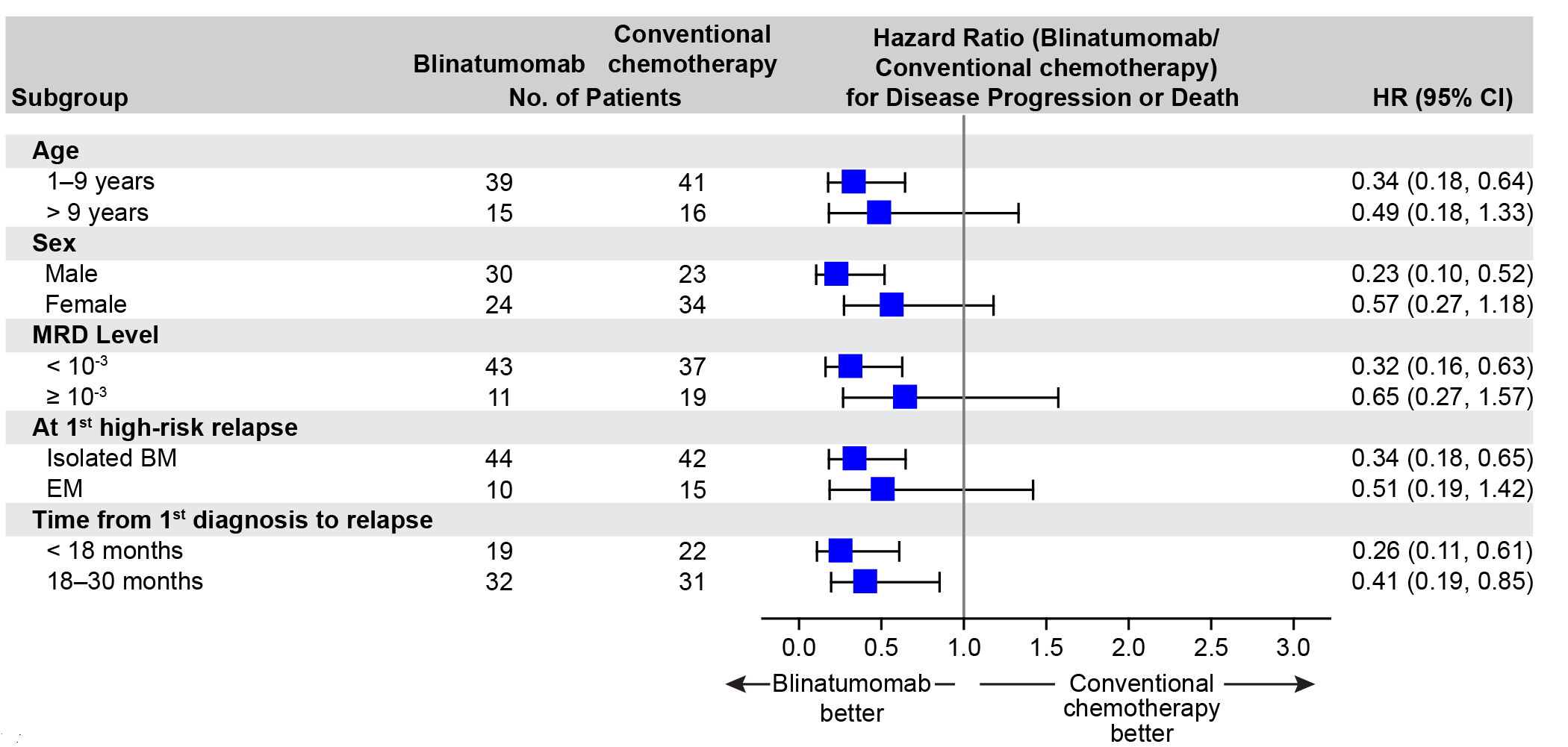


BM, bone marrow; CI, confidence interval; ; EM, extramedullary; MRD, measurable residual disease.

**Supplementary Figure 2. Kaplan-Meier estimates for relapse-free survival**


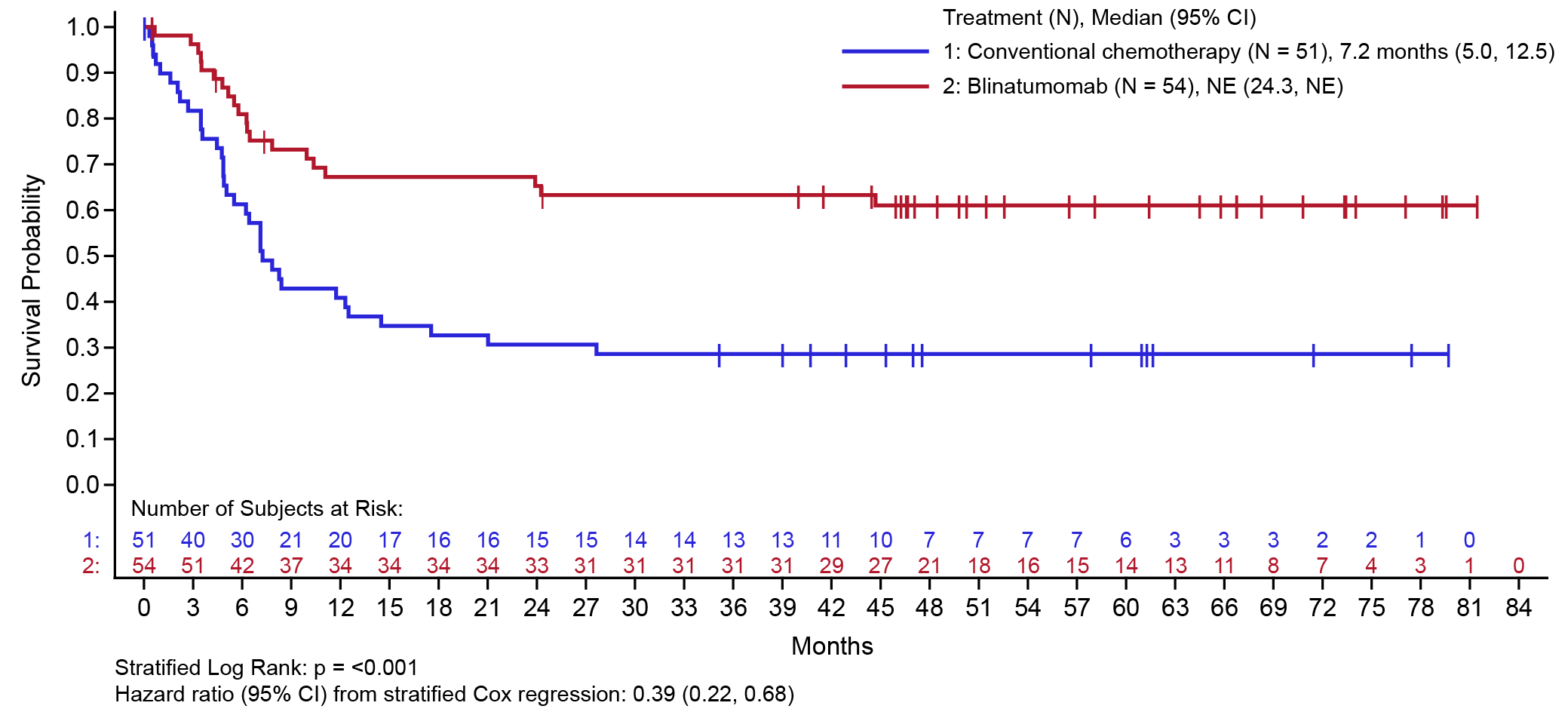


B-ALL, B-cell acute lymphoblastic leukemia; CI, confidence interval; NE, not estimable.

**Supplementary Figure 3. Subgroup analysis for overall survival outcomes**


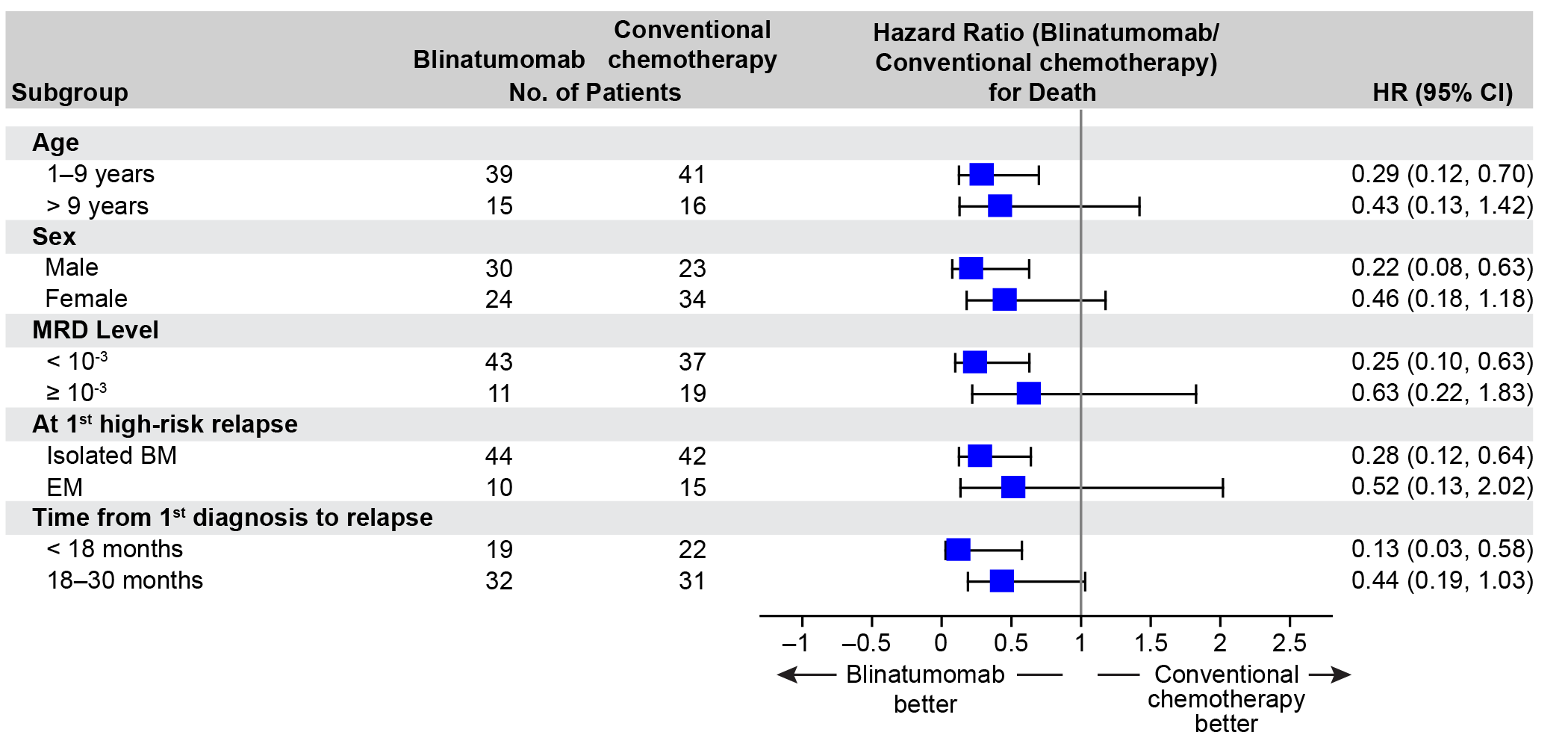


BM, bone marrow; CI, confidence interval; ; EM, extramedullary; MRD, measurable residual disease.
